# Constitutive upregulation of the transforming growth factor-β pathway in rheumatoid arthritis synovial fibroblasts

**DOI:** 10.1186/ar2217

**Published:** 2007-06-26

**Authors:** Dirk Pohlers, Andreas Beyer, Dirk Koczan, Thomas Wilhelm, Hans-Jürgen Thiesen, Raimund W Kinne

**Affiliations:** 1 Experimental Rheumatology Unit, Department of Orthopedics, Friedrich Schiller University Jena, Eisenberg, Germany; 2 Leibniz Institute for Age Research, Fritz Lipmann Institute, Beutenbergstraße 11, Jena, D-07745, Germany; 3 BIOTEC, Technical University of Dresden, Dresden, 01602, Germany; 4 Institute of Immunology, University of Rostock, Schillingallee 69, Rostock, D-18055, Germany; 5 Institute of Food Research, Colney Lane, Colney, Norwich, NR4 7UA, UK

## Abstract

Genome-wide gene expression was comparatively investigated in early-passage rheumatoid arthritis (RA) and osteoarthritis (OA) synovial fibroblasts (SFBs; *n *= 6 each) using oligonucleotide microarrays; mRNA/protein data were validated by quantitative PCR (qPCR) and western blotting and immunohistochemistry, respectively. Gene set enrichment analysis (GSEA) of the microarray data suggested constitutive upregulation of components of the transforming growth factor (TGF)-β pathway in RA SFBs, with 2 hits in the top 30 regulated pathways. The growth factor TGF-β1, its receptor TGFBR1, the TGF-β binding proteins LTBP1/2, the TGF-β-releasing thrombospondin 1 (THBS1), the negative effector SkiL, and the smad-associated molecule SARA were upregulated in RA SFBs compared to OA SFBs, whereas TGF-β2 was downregulated. Upregulation of TGF-β1 and THBS1 mRNA (both positively correlated with clinical markers of disease activity/severity) and downregulation of TGF-β2 mRNA in RA SFBs were confirmed by qPCR. TGFBR1 mRNA (only numerically upregulated in RA SFBs) and SkiL mRNA were not differentially expressed. At the protein level, TGF-β1 showed a slightly higher expression, and the signal-transducing TGFBR1 and the TGF-β-activating THBS1 a significantly higher expression in RA SFBs than in OA SFBs. Consistent with the upregulated TGF-β pathway in RA SFBs, stimulation with TGF-β1 resulted in a significantly enhanced expression of matrix-metalloproteinase (MMP)-11 mRNA and protein in RA SFBs, but not in OA SFBs. In conclusion, RA SFBs show broad, constitutive alterations of the TGF-β pathway. The abundance of TGF-β, in conjunction with an augmented mRNA and/or protein expression of TGF-β-releasing THBS1 and TGFBR1, suggests a pathogenetic role of TGF-β-induced effects on SFBs in RA, for example, the augmentation of MMP-mediated matrix degradation/remodeling.

## Introduction

Human rheumatoid arthritis (RA) is characterized by chronic inflammation and destruction of multiple joints, perpetuated by an invasive pannus tissue. Activated synovial fibroblasts (SFBs), whether irreversibly altered [1] or reversibly stimulated by the inflammatory microenvironment [2], are major components of the pannus and contribute to joint destruction by secretion of pro-inflammatory cytokines and tissue-degrading enzymes [3].

Recently, microarray techniques employing hybridization of biological samples to immobilized cDNA probes or oligonucleotide probe sets (for example, Affymetrix^®^) have been increasingly used to study genome-wide gene expression profiles and to perform initial screening for genes of potential pathogenetic interest. In the meantime, there are some studies available of differential gene expression between RA and osteoarthritis (OA) synovial membranes (SMs) [4-6], RA and OA SFBs [7] or about the effects of mediators with a central role in RA, for example, tumor necrosis factor-α and IL-1β, on SFBs [8-10]. In order to identify sets of constitutively regulated genes that can be classified into well-known pathways, differential gene expression between early passage RA and OA SFBs was investigated using Affymetrix^® ^oligonucleotide arrays and analyzed using the Gene Set Enrichment Analysis (GSEA) tool [11]. The differential expression of such pathway components in RA and OA SFBs may then indicate a more pronounced potency for further activation by the respective cytokines or growth factors, for example, transforming growth factor (TGF)-β. To enhance the significance of the array analysis, the mRNA data of the most important molecules were validated by real-time reverse transcriptase (RT)-PCR and the respective proteins were analyzed by western blots or immunohistochemistry. In addition, stimulation of SFBs with TGF-β1 was performed to prove the functional relevance of the enhanced expression of TGF-β pathway-related molecules in RA.

## Materials and methods

### Patients and samples

Synovial tissue was obtained from open joint replacement surgery or arthroscopic synovectomy at the Clinic of Orthopedics, Waldkrankenhaus "Rudolf Elle" (Eisenberg, Germany). Patients with RA or OA (*n *= 6 each for gene expression analysis and further patients for validation experiments; total of 7 RA and 9 OA patients) were classified according to the American Rheumatology Association (ARA, now American College of Rheumatology (ACR)) criteria [12] (Table 1). SFBs were purified from synovial tissue as previously published [13]. Briefly, the tissue samples were minced, digested with trypsin/collagenase P, and the resulting single cell suspension cultured for seven days. Non-adherent cells were removed by medium exchange. SFBs were then negatively purified using Dynabeads^® ^M-450 CD14 and subsequently cultured over 2 passages in DMEM containing 100 μg/ml gentamycin, 100 μg/ml penicillin/streptomycin, 20 mM HEPES and 10% FCS (all from PAA Laboratories, Cölbe, Germany).

**Table 1:** Clinical data of patients

Patient	Gender/age (years)	Disease duration (years)	RF	ESR (mm/h)	CRP (mg/ml)	No. of ARA criteria	Concurrent treatment
**Rheumatoid arthritis**							
EB 73	M/68	8	+	90	48.1	6	MTX
EB 74	F/71	17	+	45	26.9	6	NSAIDs
EB 87	F/65	12	+	50	106.7	5	NSAIDs
EB 88	F/62	10	+	90	169.5	6	NSAIDs
EB 96	M/67	4	+	68	75.7	4	NSAIDs
EB 108	F/72	45	+	70	70.7	7	NSAIDs, steroids
EB 141	F/73	1	+	57	14.3	5	NSAIDs, steroids
							
**Osteoarthritis**							
EB 77	F/66	5	-	2	<5.0	0	None
EB 81	F/56	3	-	14	<5.0	0	None
EB 90	F/61	2	-	18	<5.0	0	None
EB 102	F/73	8	-	20	<5.0	0	NSAIDs
EB 115	F/56	3	-	11	8.2	0	NSAIDs
EB 118	M/72	2	-	4	9.3	0	NSAIDs
EB 135	M/57	4	-	12	<5.0	0	NSAIDs
EB 165	F/63	3	-	11	6.1	0	NSAIDs
EB 172	M/61	3	-	5	<5.0	0	NSAIDs

ARA, American Rheumatism Association (now American College of Rheumatology); CRP, C-reactive protein (normal range <5 mg/l); ESR, erythrocyte sedimentation rate; F, female; M, male; MTX, methotrexate; NSAIDs, non steroidal anti-inflammatory drugs; RF, rheumatoid factor; -, negative; +, positive.

### Culturing of cells and isolation of total RNA

At the end of the 2nd passage, the SFBs were starved with medium containing 1% FCS for 72 h to minimize stimulating effects by serum components. After washing with PBS, the cells were lysed with RLT buffer (Qiagen, Hilden, Germany) and frozen at -70°C. Total RNA was isolated using the RNeasy Kit (Qiagen) according to the supplier's recommendation.

### Microarray data analysis

RNA probes were labeled according to the supplier's instructions (Affymetrix^®^, Santa Clara, CA, USA). Analysis of gene expression was carried out using U95A oligonucleotide arrays. Hybridization and washing of gene chips was performed according to the supplier's instructions and microarrays were analyzed by laser scanning (Hewlett-Packard Gene Scanner). Background-corrected signal intensities were determined using the MAS 5.0 software (Affymetrix^®^). Subsequently, signal intensities were normalized among arrays to facilitate comparisons between different patients. For this purpose, arrays were grouped according to patient groups (OA versus RA, *n *= 6 each). The arrays in each group were normalized using quantile normalization [14]. Original data from microarray analysis have been deposited in NCBIs Gene Expression Omnibus [15] and are accessible through GEO series accession number GSE7669.

### Gene set enrichment analysis

GSEA was performed using the software described in [11]. Briefly, GSEA searches for the enrichment of up- or downregulated genes in pre-defined pathways and subsequently performs a correction for multiple-hypotheses testing. Pathways were ranked with respect to the score values (normalized enrichment scores), which indicate differential expression. GSEA was run with default settings by performing 500 random mutations for the determination of statistical significance. The pre-defined pathways contained two variants of the TGF-β pathway (called 'TGF_Beta_Signaling_Pathway' and 'tgfbPathway'). Both pathways were among the top 30 ranking pathways (out of 259). A merged pathway was created by combining the genes from the two pre-defined pathways (TGF_joint) and GSEA was re-run including this new pathway.

### Quantitative real-time PCR analysis

cDNA was prepared from total RNA using oligo-dT primers and SuperScript reverse transcriptase (Invitrogen, Karlsruhe, Germany). For the genes of interest and the housekeeping aldolase gene, specific mRNA sequences were cloned using the TOPO-TA cloning kit (Invitrogen) and employed for the generation of external standard curves. Real-time PCR was performed on a LightCycler^® ^(Roche Diagnostics, Mannheim, Germany) using LightCycler^® ^FastStart DNA Master SYBR Green I (Roche) as previously described [16] with the primer pairs presented in Table 2. The amount of cDNA in each sample was normalized using the expression of the housekeeping aldolase gene, which showed the lowest variability over all oligonucleotide arrays. The general amplification protocol (50 cycles) was set as follows: initial denaturation for 3 minutes at 95°C; denaturation for 5 s at 95°C; specific primer annealing temperature for 10 s; amplification at 72°C for the indicated time period (Table 2). The general settings for the melting curve protocol (1 cycle) were as follows: denaturation at 95°C; cooling to 5°C above the primer annealing temperature; heating to 95°C (speed 0.1°C/s); final cooling for 5 minutes at 40°C. The fluorescence emitted by double-stranded DNA-bound SYBR-Green was measured once at the end of each additional heating step and continuously during the melting curve program. The concentrations of cDNA present in each sample were calculated by the LightCycler^®^-software using the external standard curves. Product specificity was confirmed by melting curve analysis and initial cycle sequencing of the PCR products.

**Table 2:** Primer and product sizes of real-time PCR validated genes

Gene product	Forward primer (5'→3')	Reverse primer (5'→3')	Size (bp)	T_A_/t_amp_
Aldolase	TCATCCTCTTCCATgAGACACTCTA	ATTCTgCTggCAgATACTggCATAA	314	58°C/30 s
TGF-β1	gTTCAAgCAgAGTACACACAgC	gTATTTCTggTACAgCTCCACg	157	60°C/20 s
TGF-β2	ATgCggCCTATTgCTTTAgA	TAAgCTCAggACCCTgCTgT	185	60°C/20 s
TGF-β3	CAgggAgAAAATCCAggTCA	CCTggAAggCgTCTAACCAAg	179	58°C/20 s
THBS1	gATCCTggACTCgCTgTAgg	CCgAgTATCCCTgAgCCCTC	202	60°C/20 s
TGFBR1	ATCACCTggCCTTggTCCTgTgg	GgTCCTCTTCATTTggCACTCgATg	140	54°C/20 s
SkiL	CAgTggAAACTgATggAgAgC	ggAAgAggCAgAAATACAgTAgg	193	55°C/20 s
MMP-11	ggTgTACgACggTgAAAAgCC	CAgggTCAAACTTCCAgTAgAgg	353	64°C/30 s

Bp, base-pairs; T_A_, annealing temperature; t_ampl_, amplification time.

### Western blotting

SFBs from RA patients (*n *= 6 for TGF-β1; *n *= 5 for TGF-β receptor (TGFBR)) and OA patients (*n *= 4) were cultured and starved as above. Cell lysis was performed after washing with PBS using NP-40 lysis buffer (50 mM Tris/HCl, pH 7.4, 150 mM NaCl, 1 mM EDTA, 1% NP-40, 1 mM phenylmethylsulfonylfluoride (PMSF), 1 mM Na_3_VO_4_, as well as 1 μg/ml of aprotinin, leupeptin, and pepstatin). The protein content was determined using the BCA assay (Pierce, Rockford, IL, USA) following acetone precipitation of a 25 μl sample aliquot. Proteins were resolved by reducing SDS-PAGE of 40 μg lysate and subsequently detected by immunoblotting, using the following primary antibodies: anti-TGF-β1 (A75-2, BD Biosciences, Heidelberg, Germany), anti-TGFBR 1 (#3712, CellSignal, Beverly, MA, USA), as well as goat anti-mouse IgG horseradish peroxidase (HRP; A-3682, Sigma-Aldrich, Steinheim, Germany) or goat anti-rabbit IgG HRP (sc-2004, St Cruz Biotechnology, Heidelberg, Germany) as secondary antibodies. The blots were then stripped and re-probed with mouse anti-human β-actin (clone AC-15, Sigma-Aldrich, Deisenhofen, Germany) and goat anti-mouse IgG HRP to ensure equal loading. In the case of TGF-β1, attempts to quantify protein levels by ELISA in the supernatants of cultured SFBs were not successful, possibly due to its association with the extracellular matrix surrounding the cells.

### Immunohistochemistry

SFBs from RA and OA patients (*n *= 3 each) were cultured and starved in chamber slides (10^4 ^cells per well) as above. After washing with PBS and fixing in 10% formalin in PBS for 10 minutes, the antigen was unmasked by treating the cells with citrate buffer (10 mM; pH 6.0 with NaOH, 0.05% Tween20) and heating for 5 minutes in a microwave oven (300 W). After cooling and washing with PBS, the slides were blocked with 5% goat serum in PBS for 30 minutes, followed by incubation for 30 minutes with the primary antibody (mouse anti-human thrombospondin (THBS)1, clone A6.1, LabVision c/o Dunn Labortechnik, Asbach, Germany) diluted at 4 μg/ml in 1% goat serum. HRP-conjugated rabbit anti-mouse IgG (in PBS/1% goat serum) was added for 30 minutes. The peroxidase was revealed using diaminobenzidine for 5 minutes, and the slides were washed and covered with Aquatex (Merck, Darmstadt, Germany). A mouse IgG_1 _monoclonal antibody (MOPC21, Sigma; 4 μg/ml) served as control and yielded negative results. Positively stained cells were scored semi-quantitatively by two observers (DP and RWK) in a blinded manner (0 = no; 1 = weak; 2 = medium; 3 = strong staining).

### Stimulation with TGF-β1

SFBs from RA patients (*n *= 3) and OA patients (*n *= 4) were cultured and starved as above. Recombinant human TGF-β1 (Peprotech, London, UK) was added at 10 ng/ml for 4 h. After washing and lysing, RNA isolation, cDNA synthesis, and quantitative real-time PCR (qPCR) for aldolase and matrix-metalloproteinase (MMP)-11 were performed as described above. Protein expression of MMP-11 was assessed by intracellular staining of stimulated cells (10 ng/ml TGF-β1, 48 h) by flow cytometry on a FACScan cytometer (BD, Heidelberg, Germany). The cells were trypsinized, washed with PBS/1% FCS and fixed with 4% paraformaldehyde in PBS for 15 minutes at 4°C. After permeabilization with 0.5% saponin in PBS/1% FCS, the cells were incubated with anti-human-MMP-11 antibody (clone 135421, R&D Systems, Wiesbaden, Germany), followed by goat anti-mouse IgG FITC (Dako, Hamburg, Germany). A mouse-anti-keyhole limpet hemocyanin (KLH) antibody (IgG_2b_, clone 20116, R&D Systems) served as isotype control.

### Statistical analysis

The non-parametric Mann-Whitney *U *test was applied for the comparison of differences between RA and OA in qPCR, western blots, immunohistochemistry, and flow cytometry assays. Statistically significant differences were accepted for *p *≤ 0.05. For correlations between gene expression and clinical parameters, the Spearman Rank Test was used (*p *≤ 0.01).

## Results

### Comparison of constitutive gene expression in RA and OA SFB by GSEA

**Figure 1:**
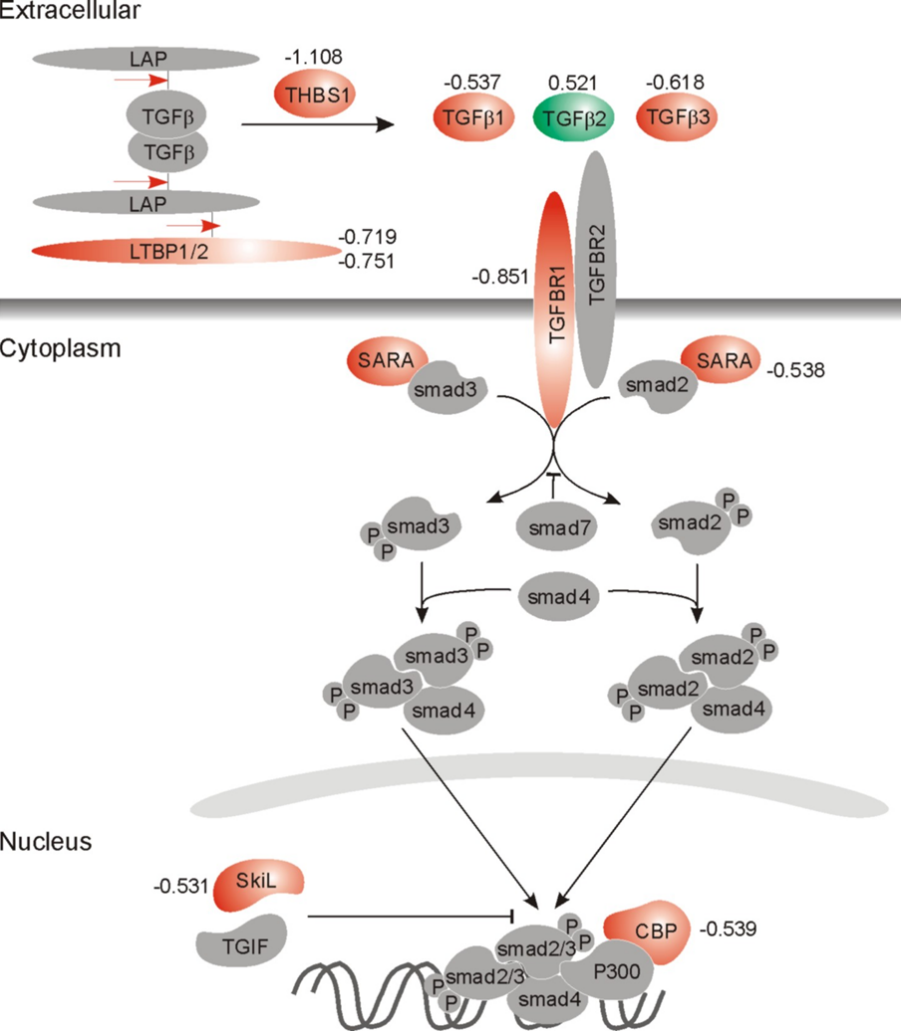
Central components of the transforming growth factor (TGF)-β pathway are shown with their scores, as determined by Gene Set Enrichment Analysis of the oligonucleotide microarray data. Molecules upregulated in synovial fibroblast (SFBs) from rheumatoid arthritis patients are shown in red (negative values), and those upregulated in osteoarthritis SFBs are shown in green (positive values). Red arrow indicates the cleavage site. CBP, CREB binding protein; LAP, latency-associated protein; LTBP, latent TGF-β binding protein; P, phosphate; TGFBR, TGF-β receptor; TGIF, TGFB-induced factor; THBS, thrombospondin.

Gene expression in early passage SFBs derived from SM of 6 RA patients was compared to that in SFBs from SM of 6 OA patients. The GSEA software was used to evaluate the gene expression values and to classify the data into various pathways depending on the augmented expression of pathway-related genes. Out of 259 pathways, 96 pathways were upregulated and 163 pathways were downregulated in RA SFBs compared to OA SFBs. The TGF_Beta_Signaling_Pathway was ranked sixth after five other pathways (for example, Inflammatory_Response_Pathway, CR_Immune_Function, IL7_Pathway) for upregulation in RA SFBs (Table 3). Another variant of the TGF-β pathway was also among the top 30 pathways (26th place), as was the 'joint pathway' (TGF_joint, 8th place), which was created by merging the genes from the two pathways (see Materials and methods). In addition, GSEA was applied to compare the gene expression values of OA and RA SFBs and to score each gene according to the mean value in the respective group. Marginal differences were excluded by considering only scores higher than 0.4 or less than -0.4. Positive values indicate lower expression in RA SFBs, and negative values show more pronounced expression in RA SFBs. For the TGF-β pathway, the most important components are selected and listed in Table 4. The function of the molecules within the pathway in conjunction with their scores is demonstrated in Figure 1.

**Table 3:** Top 30 differentially regulated pathways in rheumatoid arthritis using Gene Set Enrichment Analysis

Rank	Name	Size^a^	NES
1	Inflammatory_Response_Pathway	42	-1.7343
2	CR_IMMUNE_FUNCTION	72	-1.4860
3	cell_motility	148	-1.3477
4	il7Pathway	37	-1.3376
5	breast_cancer_estrogen_signalling	167	-1.2575
6	TGF_Beta_Signaling_Pathway	76	-1.2538
7	p38mapkPathway	72	-1.2517
8	TGF_joint	91	-1.2494
9	ST_Ga12_Pathway	36	-1.2444
10	P53_UP	55	-1.2301
11	SA_CASPASE_CASCADE	31	-1.2147
12	Wnt_Signaling	79	-1.2121
13	fasPathway	60	-1.1990
14	EMT_DOWN	56	-1.1655
15	CR_CAM	143	-1.1651
16	cell_adhesion	245	-1.1628
17	NFKB_REDUCED	30	-1.1577
18	mRNA_processing	55	-1.1485
19	ST_MONOCYTE_AD_PATHWAY	39	-1.1485
20	GPCRs_Class_B_Secretin-like	28	-1.1401
21	GLUCOSE_DOWN	261	-1.1364
22	tnf_and_fas_network	33	-1.1302
23	ST_Fas_Signaling_Pathway	97	-1.1263
24	shh_lisa	33	-1.1164
25	intrinsicPathway	28	-1.1160
26	tgfbPathway	35	-1.0937
27	alkPathway	57	-1.0871
28	hdacPathway	45	-1.0860
29	41bbPathway	33	-1.0787
30	ST_Ga13_Pathway	54	-1.0367

^a^Size refers to the number of included genes. NES, normalized enrichment scores

**Table 4:** GSEA scores of differentially regulated genes of the TGF-β pathway

Probe set	Gene symbol^a^	Gene name^a^	Score^b^
1262_s_at/971_s_at	TGFB2	Transforming growth factor, beta 2	0.521/0.404
1865_at	SKIL	SKI-like	-0.480
1830_s_at/1634_s_at	TGFB1	Transforming growth factor, beta 1	-0.529/-0.537
1866_g_at	SKIL	SKI-like	-0.531
38889_at	ZFYVE9	Zinc finger, FYVE domain containing 9 (SARA)	-0.538
33831_at	CREBBP	CREB binding protein, CBP	-0.539
38311_at	TGIF2	TGFB-induced factor 2	-0.559
1734_at	TGFB3	Transforming growth factor, beta 3	-0.618
1495_at	LTBP1	Latent TGFB binding protein 1	-0.719
37906_at	LTBP2	Latent TGFB binding protein 2	-0.751
1957_s_at/32903_at	TGFBR1	Transforming growth factor, beta receptor I	-0.778/-0.851
867_s_at/866_at/115_at	THBS1	Thrombospondin 1	-0.936/-1.104/-1.108

^a^Official gene symbol and gene name approved by the HUGO Gene Nomenclature Committee (HGNC). ^b^Calculated by Gene Set Enrichment Analysis (GSEA) software from the mean expression value.

The mRNA for TGF-β1 and TGF-β3, TGFBR1, the latent TGF-β binding proteins 1/2 (LTBP1/2), the TGF-β-releasing THBS1, the TGF-β induced factor 2 (TGIF2), the CREB binding protein (CREBBP), the SKI-like protein (SKIL), as well as the smad-associated molecule SARA (smad anchor of receptor activation; ZFYVE9) were upregulated in RA SFBs when compared to OA SFBs, with scores between -0.48 and -1.108 (Table 4). Interestingly, a positive score was observed for TGF-β2 (0.521), indicating augmented expression in OA SFBs compared to RA SFBs.

### Quantitative PCR analyses of differentially expressed genes

**Figure 2:**
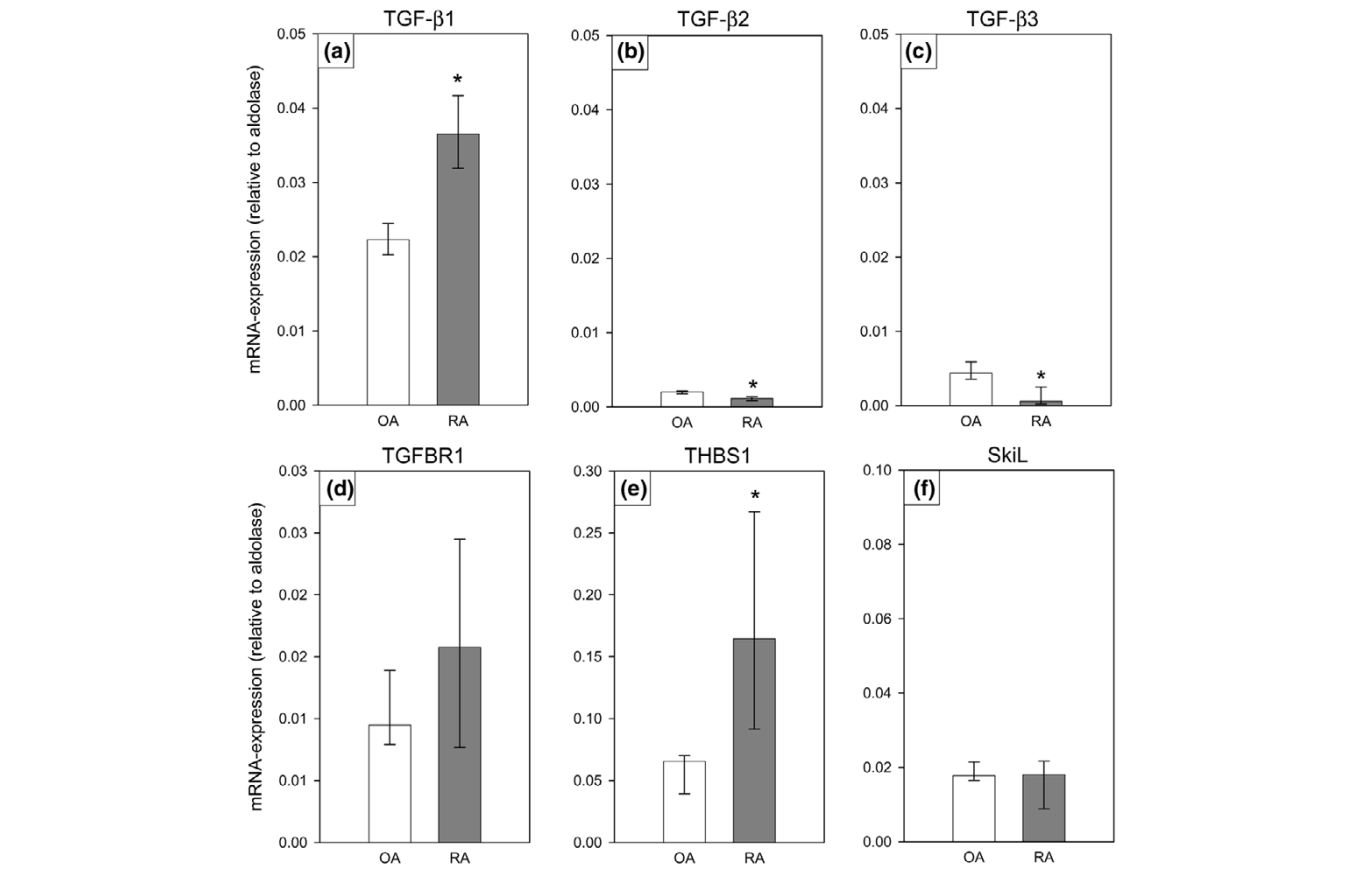
mRNA-expression of the transforming growth factor (TGF)-β related genes: **(a) **TGF-β1, **(b) **TGF-β2, **(c) **TGF-β3, **(d) **TGF-β receptor 1 (TGFBR1), **(e) **thrombospondin 1 (THBS1), and **(f) **skiL in osteoarthritis (OA) synovial fibroblast (SFBs) and rheumatoid arthritis (RA) SFBs (*n *= 6 each), as assessed by quantitative real-time PCR. Bars indicate the medians ± 75th and 25th percentiles relative to the expression of aldolase. *p ≤ 0.05 compared to OA.

To validate the results of the array analysis, differentially expressed genes of the TGF-β pathway were analyzed by independent qPCR. A significantly higher, constitutive expression of TGF-β1 (Figure 2a) and a significantly lower expression of TGF-β2 (Figure 2b) in RA SFBs versus OA SFBs was confirmed by qPCR. In contrast to the array data, the mRNA for TGF-β3 was significantly downregulated in RA SFBs. However, as in the case of TGF-β2, the relative levels of TGF-β3 (0.0001 to 0.002) were generally low compared to those of TGF-β1 (0.02 to 0.04).

The upregulation of TGF-β1 mRNA was accompanied by significantly higher expression of the TGF-β-releasing factor THBS1 in RA SFBs versus OA SFBs (Figure 2e). The constitutively increased expression of the TGFBR1 (only numerically increased) and the SkiL gene by RA SFBs in comparison to OA SFBs was not confirmed by qPCR (Figure 2d,f).

### Protein expression of TGF-β pathway-related molecules

To further validate the constitutive elevation of the TGF-β pathway in early passage RA SFBs, the translation of mRNA into proteins was analyzed in SFBs from additional OA and RA patients by western blots for TGF-β1 and TGFBR1, as well as immunohistochemistry staining for THBS1.

**Figure 3:**
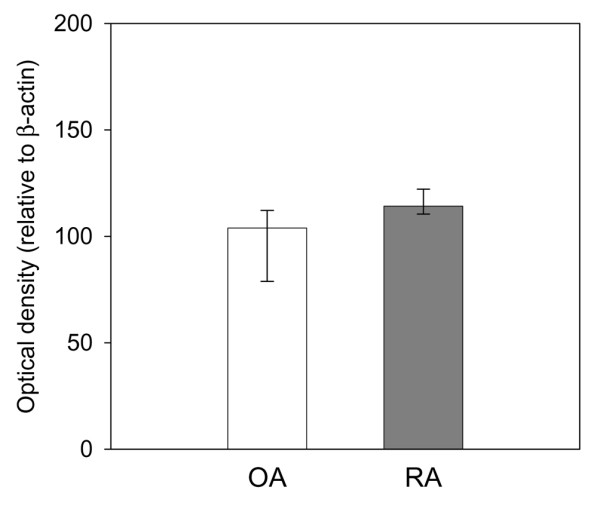
Transforming growth factor (TGF)-β1 protein expression in osteoarthritis (OA) synovial fibroblast (SFBs) (*n *= 4) and rheumatoid arthritis (RA) SFBs (*n *= 6), as assessed by SDS-PAGE/western blotting. Bars indicate the optical density of the protein bands (median ± 75th and 25th percentiles) relative to the β-actin control.

**Figure 4:**
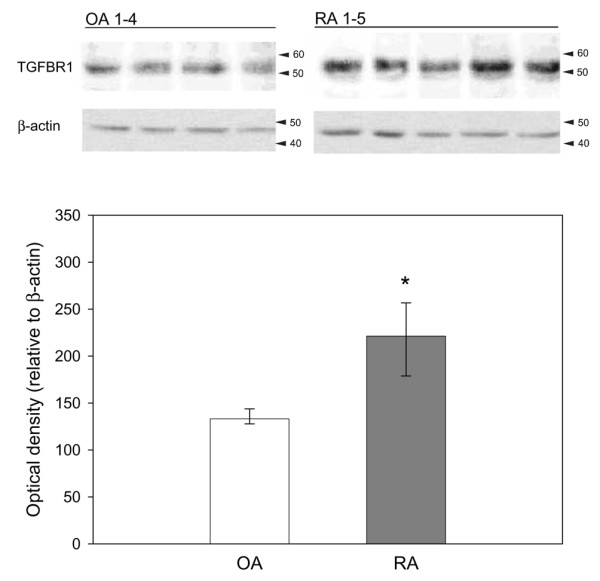
TGF-β receptor 1 (TGFBR1) and β-actin (control) protein expression in osteoarthritis (OA) synovial fibroblast (SFBs) (*n *= 4) and rheumatoid arthritis (RA) SFBs (*n *= 5) as assessed by SDS-PAGE/western blotting (upper panel). Bars indicate the median optical density of protein bands ± 75th and 25th percentiles relative to the value of the β-actin bands (lower panel). *p ≤ 0.05 compared to OA.

In contrast to the mRNA expression data by array hybridization and real-time PCR data, the protein for TGF-β1 was only numerically increased in RA SFBs (Figure 3). The protein levels of TGFBR1, the receptor that transduces the signal into the cell after binding of TGF-β (Figure 1), were significantly upregulated in RA SFBs compared to OA SFBs (Figure 4; blot and quantification).

**Figure 5:**
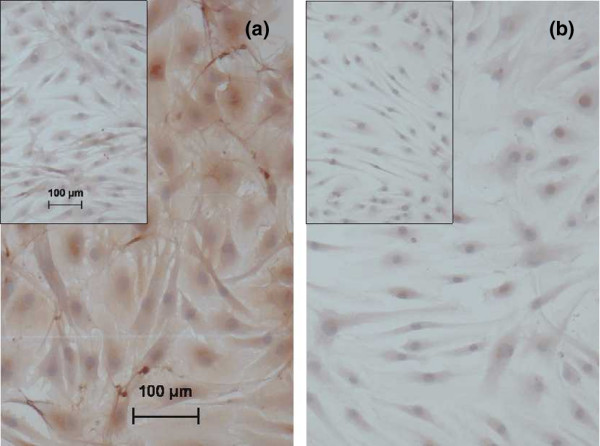
Protein expression of thrombospondin 1 (THBS1) in **(a) **rheumatoid arthritis (RA) synovial fibroblast (SFBs) and osteoarthritis SFBs **(b)**, as assessed by immunohistochemistry. Brown staining indicates the presence of THBS1 in SFBs from one representative of three RA and three OA patients; nuclei are counterstained in blue (hematoxylin). The respective staining with an isotype control instead of the specific primary antibody is demonstrated in the insert; the magnification scale is shown in (a).

The protein expression of THBS1, known to activate TGF-β by releasing it from the latent form, was investigated by immunohistochemistry and semi-quantitative scoring methods. Cultured RA SFBs showed a significantly stronger staining for THBS1 (2.33 ± 0.33 (mean ± standard error of the mean)) than OA SFBs (0.66 ± 0.33; *p *= 0.02). Representative staining for THBS1 and the respective isotype antibody in SFBs from one patient each with RA or OA is shown in Figure 5.

### Stimulation of SFBs with TGF-β1

**Figure 6:**
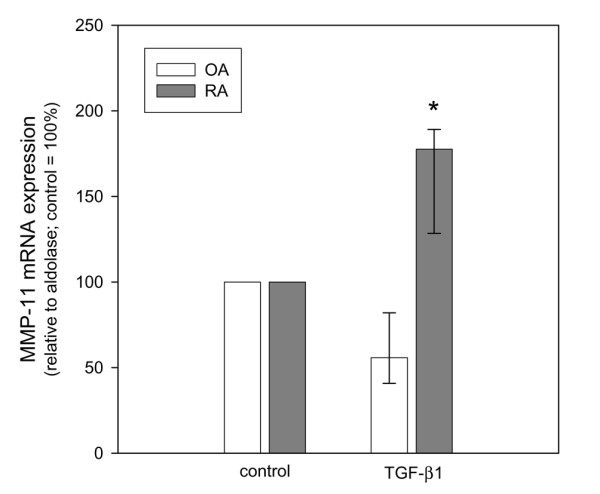
Transforming growth factor (TGF)-β1-stimulated mRNA expression for matrix-metalloprotease (MMP)-11 in osteoarthritis (OA) synovial fibroblast (SFBs) (*n *= 4) and rheumatoid arthritis (RA) SFBs (*n *= 3), as assessed by qPCR. Bars indicate the medians ± 75th and 25th percentiles relative to the expression of aldolase, expressed as percent of the unstimulated control (= 100%). *p ≤ 0.05 compared to OA.

**Figure 7:**
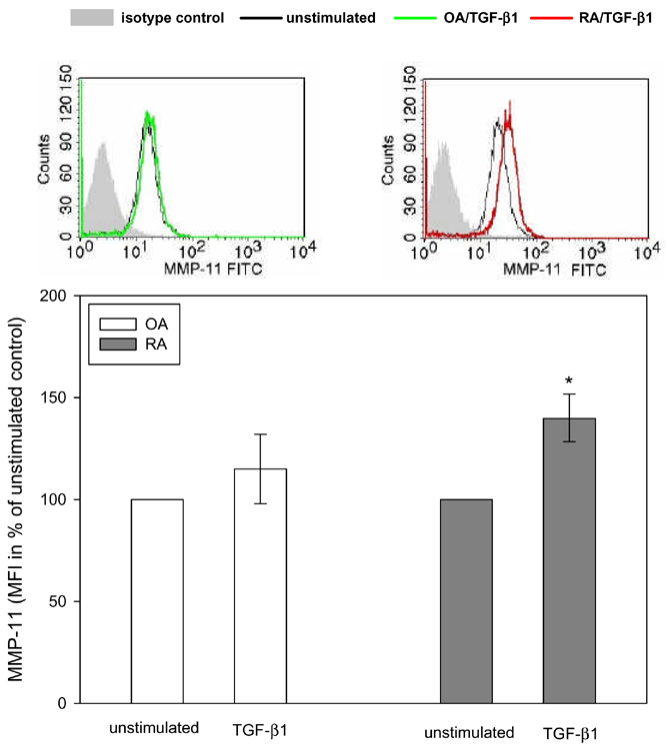
Transforming growth factor (TGF)-β1-stimulated intracellular protein expression for MMP-11 in osteoarthritis (OA) synovial fibroblast (SFBs) (*n *= 5) and rheumatoid arthritis (RA) SFBs (*n *= 4), as assessed by flow cytometry. Representative histograms from one RA and OA patient are shown following TGF-β1 stimulation for 48 h (upper panel) and the medians ± 75th and 25th percentiles of the mean fluorescence intensities are expressed for all patients as percent of the unstimulated control (= 100%; lower panel). *p ≤ 0.05 compared to OA.

In order to address the functional relevance of the constitutively activated TGF-β pathway, SFBs from RA and OA patients were stimulated with recombinant TGF-β1 and the effect on gene expression for MMP-11 (stromelysin 3) was analyzed by qPCR. A significantly enhanced MMP-11 mRNA expression was observed 4 h after stimulation with TGF-β1 in RA SFBs, but not in OA SFBs (Figure 6). In addition, intracellular MMP-11 protein, measured as mean fluorescence intensity of MMP-11 positive cells, increased in RA SFBs to significantly higher levels than in OA SFBs (Figure 7). However, the proliferation response of SFBs to TGF-β1 was not different (data not shown).

### Correlation with clinical parameters

Analyzing RA and OA SFBs together, significant positive correlations were observed between the constitutive expression of TGF-β1 mRNA (but not protein) and the serum levels of C-reactive protein (r = 0.711, *n *= 12; *p *= 0.01), as well as the number of fulfilled American Rheumatism Association (now American College of Rheumatology) criteria (r = 0.726, *n *= 12; *p *= 0.007). The latter also correlated significantly with the constitutive THBS1 mRNA expression (r = 0.726, *n *= 12; *p *= 0.007).

## Discussion

The aim of the present study was to systemically analyze differentially expressed pathways in purified, early passage SFBs derived from RA and OA patients. For this purpose, gene expression was measured with oligonucleotide array technology and validated by other, low-throughput methods.

SFBs showed a differential, constitutive expression of genes involved in various cellular pathways (Table 3). Using the software tool GSEA for pathway scanning [11], components of the TGF-β pathway were found to be over-represented among these genes. As well as TGF-β1 and its receptor TGFBRI, the following molecules were also upregulated in RA SFBs: LTBP1 and LTBP2, both components of the large latent TGF-β complex binding TGF-β to the extracellular matrix [17]; THBS1, known to release active TGF-β from its latent form [18]; and SARA, which recruits the TGF-β-signal-transducing smads to the membrane in the close vicinity of the receptor [19] (Figure 1). This interesting finding provides detailed analysis of individual components of the TGF-β pathway and parallels recent reports on the existence of two distinct gene expression profiles in SFBs, one of which is characterized by the expression of TGF-β/activin A-inducible genes [7]. Although hierarchical clustering of the data in the present study did not reveal such distinct profiles in purified SFBs (data not shown; possibly due to the low number of samples), the overexpression of TGF-β-related genes supports the importance of this pathway in synovial pathology. This is further underlined by significant correlations between the constitutive TGF-β1 and THBS1 mRNA expression in SFBs and the C-reactive protein levels or the number of fulfilled ARA criteria, that is, clinical markers of disease activity and/or severity.

TGF-β1 mRNA was expressed to a significantly higher degree in RA SFBs than OA SFBs, in parallel with previous reports showing a significantly higher expression of TGF-β1 in the RA SM than in the OA SM [20-22]. Specific assignment of TGF-β1 production to fibroblast-like synoviocytes in the RA SM [21] or to fibroblasts in synovial regions with pronounced fibrosis provides evidence for a pro-fibrotic role of TGF-β1 in RA [22]. Also, the expression of TGF-β1 directly at the cartilage-pannus junction during the most severe phase of rat collagen-induced arthritis [23] suggests TGF-β1 has an important pro-destructive role in experimental arthritis.

The observed discrepancy between the expression of TGF-β1 mRNA and protein in SFBs may be due to the fact that blot analysis with the present monoclonal antibody underestimates the total amount of protein by detecting only the active form of TGF-β1. In fact, newly synthesized TGF-β1 is predominantly secreted as the latent proform, as also observed in irradiated rat mesangial cells [24]. On the other hand, known post-transcriptional regulation of the TGF-β1 gene *via *the 5' untranslated region may prevent its proportional translation into protein [25]. Secretion of TGF-β1 into the supernatant of the cells was excluded as a possible reason for the observed discrepancy, since no signal was obtained by ELISA.

In contrast to the levels of TGF-β1 mRNA (upregulated in RA SFBs), the amounts of TGF-β2 and TGF-β3 mRNA, which share the same receptors, were significantly increased in OA SFBs by qPCR. This is in agreement with results showing on the one hand predominant effects of TGF-β2/3 versus TGF-β1 in an age model of OA [26], but on the other hand a strong immunoreactivity at the protein level only for TGF-β1 but not for TGF-β2/3 in the RA SM [27]. In addition, there is strong evidence that the isoforms have different functions, as demonstrated by the non-overlapping phenotypes of the isoform-specific null mice [27]. Together, these findings argue for a pivotal and differential role of TGF-β1 in the pathogenesis of RA.

In addition to TGF-β itself, its receptor TGFBRI was upregulated in RA SFBs, providing the basis for an enhanced autocrine effect of locally present TGF-β1 on SFBs in the RA SM. Whereas this study provides the first report concerning the expression of TGFBR1 in human arthritis, the type II receptor [22] and endoglin (a receptor for TGF-β1 and 3) [20] have been reported to be more strongly expressed in RA SM than in OA SM or normal SM, showing the relevance of TGF-β-signaling in RA.

The present study shows a constitutive upregulation of THBS1 (mRNA/protein) in RA SFBs. A constitutively higher expression of THBS1 has previously been described in RA synovium compared to OA and joint trauma [28] and the synovial expression of this molecule has been assigned to endothelial cells, macrophages and synovial lining cells [29]. Furthermore, it has been demonstrated that implantation of THBS1-containing pellets into the ankle joints of rats aggravates adjuvant arthritis [30], showing the importance of THBS1 in arthritis. TGF-β is initially produced in its latent form [31,32], that is, covalently linked with the latency-associated proteins and attached to LTBPs, which are cross-linked to the extracellular matrix (reviewed in [17]). In order to activate TGF-β, the mature molecule has to be released from the large latent complex by plasmin or cathepsins [33] or, as previously described, with a high efficiency by THBS1 [18]. The abundance of THBS1 may, therefore, lead to enhanced activation of latent TGF-β1 in RA (see above), resulting in more TGF-β1 activity in the arthritic joint. Indeed, increased levels of active TGF-β1 have recently been reported in RA synovial fluid in comparison to OA synovial fluid [34]. This TGF-β1 activity may then contribute to enhanced proliferation of SFBs [35] or enhanced production of MMPs [36].

In the present study, the induction of MMP-11 (stromelysin-3) at the mRNA and protein levels by TGF-β1 was restricted to RA SFBs. This effect has been originally described for mouse fibroblasts and osteoblasts and was based on both stimulation of gene transcription and stabilization of mRNA transcripts [37]. The rapid upregulation of mRNA after 4 h suggests a direct activation of gene transcription rather than an indirect induction via other factors, that is, platelet-derived growth factor [38], which is also a known inducer of MMP-11 [39]. Like other MMPs, MMP-11 requires proteolytic removal of propeptides for activation. Whereas for other MMPs this process occurs via extracellular proteases following secretion, MMP-11 is intracellularly processed by furin and secreted as an active protease (in analogy to membrane-type MMP) [40]. Therefore, the presence of increased intracellular levels observed in the present study represents the basis for functional MMP-11 outside the cell. Although MMP-11 does not directly participate in the degradation of extracellular matrix, it is able to inactivate protease inhibitors, resulting in enhanced proteolytic activity [41] and controls cell proliferation by processing the insulin-like growth factor-binding protein-1 [42]. MMP-11 is, therefore, involved in matrix turnover and proliferation, both processes with implications for RA. Its specific upregulation by TGF-β further supports a functional relevance of the constitutively upregulated TGF-β pathway in RA.

## Conclusion

The presence of TGF-β, in conjunction with augmented mRNA/protein expression of the TGF-β releasing THBS1 and higher TGFBR1 protein by RA SFBs, suggests that TGF-β-induced effects have a (autocrine) pathogenetic importance in RA, for example, the induction of MMP-mediated matrix degradation/remodeling.

## Abbreviations

ELISA = enzyme-linked immunosorbent assay; FCS = fetal calf serum; GSEA = Gene Set Enrichment Analysis; HRP = horseradish peroxidase; IL = interleukin; LTBP = latent TGF-β binding protein; OA = osteoarthritis; PBS = phosphate-buffered saline; qPCR = quantitative real-time PCR; RA = rheumatoid arthritis; SFB = synovial fibroblast; SM = synovial membrane; TGF-β = transforming growth factor beta; TGFBR = TGF-β receptor; THBS = thrombospondin.

## Competing interests

The authors declare that they have no competing interests.

## Authors' contributions

DP performed the real-time PCR, the western blots, the immunohistochemistry, as well as the respective data analyses and participated in writing the manuscript. AB analyzed the microarray data and participated in writing the manuscript. DK performed the microarray experiments, HJT and TW participated in the coordination of the study, and RWK contributed to the design of the study and participated in the layout, writing, and finalization of the manuscript.
